# *IL28B* Alleles Exert an Additive Dose Effect When Applied to HCV-HIV Coinfected Persons Undergoing Peginterferon and Ribavirin Therapy

**DOI:** 10.1371/journal.pone.0025753

**Published:** 2011-10-07

**Authors:** Barham K. Abu Dayyeh, Namrata Gupta, Kenneth E. Sherman, Paul I. W. de Bakker, Raymond T. Chung, for the Aids Clinical Trials Group A5178 Study Team

**Affiliations:** 1 Gastrointestinal Unit, Massachusetts General Hospital, Boston, Massachusetts, United States of America; 2 Department of Medicine, Harvard Medical School, Boston, Massachusetts, United States of America; 3 Program in Medical and Population Genetics, Broad Institute of MIT and Harvard, Cambridge, Massachusetts, United States of America; 4 Department of Internal Medicine, University of Cincinnati, Cincinnati, Ohio, United States of America; 5 Division of Genetics, Department of Medicine, Brigham and Women's Hospital, Harvard Medical School, Boston, Massachusetts, United States of America; 6 Julius Center for Health Sciences and Primary Care, University Medical Center, Utrecht, The Netherlands; 7 Division of Biomedical Genetics, Department of Medical Genetics, University Medical Center, Utrecht, The Netherlands; Duke University School of Medicine, United States of America

## Abstract

**Background:**

Genetic studies have demonstrated a strong association between single nucleotide polymorphisms (SNPs) at *IL28B* and response to treatment with peginterferon (PEG) and ribavirin (RBV) in HCV monoinfected persons. We sought to test these associations in a prospective PEG / weight based ribavirin (WBR) treatment trial (ACTG A5178) (National Institution of Health registration number NCT00078403) in persons with HCV-HIV coinfection, and to develop a prediction score.

**Methods:**

We selected subjects enrolled in A5178 who completed at least the first 12 weeks of the trial and had DNA available, and genotyped three SNPs at *IL28B* (rs12979860, rs12980275, rs8099917). We used multivariate logistic regression analysis to evaluate the association between *IL28B* SNPs and HCV treatment outcomes and to develop the prediction score.

**Results:**

231 HCV/HIV coinfected subjects were included. We observed a strong association between *IL28B* genotype and response to therapy among those with genotypes 1 or 4 (odds ratio for complete early virologic responses (cEVR) and sustained virologic response (SVR) was 2.98 [1.7–5.3] and 3.4 [1.7–6.9], respectively, for each additional copy of the C allele of rs12979860). Differences in frequency of the responder allele explained some of the discrepancy in HCV treatment outcomes between blacks and whites. A simple pretreatment prediction score that incorporates the *IL28B* genotype and baseline HCV viral load has a 93% negative predictive value (NPV) for SVR.

**Conclusions:**

*IL28B SNPs* have an additive allele dose effect in predicting HCV treatment outcomes in HCV/HIV coinfected persons and can be incorporated into a simple pretreatment prediction score that could minimize the risk of exposure to PEG/RBV therapy for persons with unfavorable scores.

## Introduction

Hepatitis C virus (HCV) coinfection occurs in about one third of subjects infected with the human immunodeficiency virus (HIV) in the United States and Europe, owing to similarities in viral acquisition risk factors and chronic persistence in the majority of hosts.[1], [2] Since the introduction of highly active antiretroviral therapy (HAART) and the extended survival of persons infected with HIV, the burden of HCV in coinfected persons is now fully realized, with HCV emerging as the leading non-AIDS cause of death. [3], [4], [5], [6]

Another complicating factor is that liver fibrosis progression is accelerated in coinfected persons, and response to pegylated interferon (PEG) and ribavirin (RBV) treatment is generally poor compared with HCV monoinfected persons. [7], [8] Sustained virological response rates (SVR) in coinfected persons have ranged from 27%–40%. [9], [10], [11] Potential drug interactions with HAART, higher rates of serious side effects, high cost, and poor adherence to therapy have been some of the factors explaining poor HCV treatment outcomes in coinfection. [2] To that end, pretreatment algorithms that are able to segregate coinfected subjects based on likelihood of response to this potentially harmful and expensive therapy with limited efficacy are greatly needed.

Several studies have examined viral and host factors predicting HCV virologic response in both HCV monoinfected and HIV coinfected cohorts. Significant HCV factors associated with favorable outcome include viral genotypes 2 and 3 and low baseline HCV viral load. [12] Host factors associated with favorable outcome in some studies include young age, female gender, European descent (ethnicity), absence of advanced fibrosis and cirrhosis, and initial response to PEG therapy as represented by rapid virologic (RVR) and early virologic responses (EVR), defined as clearance of HCV RNA by week 4 and reduction of HCV RNA by at least 100-fold or clearance by week 12, respectively. [12], [13], [14]

Recent genome-wide association studies (GWAS) have shown a striking association between single nucleotide polymorphisms (SNPs) around the *IL28B* gene (specifically, rs12979860, rs8099917, and rs12980275) and response to PEG/RBV HCV treatment.[15], [16], [17] *IL28B* encodes the type III interferon IFN-*λ*3, which belongs to the IFN-*λ* family, which itself has potent antiviral activity.[18], [19]

Since the original reports, many studies have confirmed these associations in both mono- and co-infected cohorts. [20], [21], [22], [23], [24], [25], [26], [27], [28], [29] Common limitations of studies of HCV/HIV coinfected cohorts, however, have been their retrospective nature and lack of ethnic diversity. Therefore, we sought to clarify the association between *IL28B* SNPs and response to PEG/RBV treatment in the prospective multiethnic Sustained Long-Term Antiviral Maintenance Therapy in HCV/HIV-Coinfected Patients (SLAM-C) cohort.[30] Furthermore, we developed a pretreatment prediction score to identify subjects with low probability to respond to peg-IFN based therapy, to minimize their unnecessary exposure to potentially harmful therapy.

## Methods

### Study design

A5178 was a multicenter prospective, randomized, open-label, controlled trial (National Institute of Health Registration number NCT00078403) funded by the National Institute of Allergy and Infectious Diseases (NIAID) and conducted by the AIDS Clinical Trials Group (ACTG) to elucidate the role of maintenance PEG therapy in HCV treatment non-responders with HIV coinfection.[30] The trial enrolled 330 subjects from August 2004 to April 2007 from 36 ACTG sites within the United States and the results of the main study have been published. [30]

A5178 included subjects coinfected with HCV (genotypes 1, 2, 3, 4) and HIV-1, who were at least 18 years old, with HIV viral load less than 50,000 copies/mL, and a CD4 count of at least 200 cells/mm^3^. All subjects had detectable HCV RNA, at least stage 1 liver fibrosis on a biopsy performed within 2 years prior to study initiation. Subjects could be either HCV treatment naive or experienced. Those with history of AIDS-defining opportunistic infections, decompensated liver disease, other liver diseases (hepatitis B, acute hepatitis A, hemochromatosis, or homozygotic alpha-1 antitrypsin deficiency), recent steroid use, active drug/alcohol abuse, uncontrolled seizure disorders, uncontrolled active depression, history of autoimmune disease, or history of major organ transplantation were excluded.

The trial was conducted in three steps. In Step 1, all subjects were treated with PEG-IFN alfa 2a (PEG) 180 mcg SQ per week plus weight based RBV (WBR) for 12 weeks to separate treatment responders from non-responders by EVR (defined as either at least a 100-fold decrease or undetectable HCV RNA [<600 IU/mL] at 12 weeks). Non-responders entered Step 2 (the main trial) and were randomized to 72 additional weeks of maintenance therapy or to observation with no treatment. Responders entered a single-arm study (Step 3) and received a total of 72 weeks of PEG and WBR therapy.

For this study, we included all subjects who completed at least Step 1 of ACTG A5178, consented for genetic testing prior to trial entry[30], and had DNA available for *IL28B* genotyping. Thus, out of 297 subjects completing Step 1, 231 were included in this study. SVR was assessed 24 weeks after treatment cessation in step 3. None of those who entered step 2 achieved SVR.

### Clinical assessments and outcomes measurement

Study visits occurred at weeks 2, 4, 8, and 12 in the first step of the trial, and at weeks 2, 4,8,12, 16, 24, 32, 40, 48, 56, 64, and 72 in steps 2 and 3 to monitor adherence and perform routine safety clinical and laboratory assessment. HCV RNA was tested using the Roche Cobas Amplicor assay with a lower detection limit of 600 IU/mL for the quantitative assay (used in Step 1) and 60 IU/mL for the qualitative assay (used in Steps 2 and 3). HIV RNA was tested at using Roche Ultrasensitive HIV reverse transcriptase–polymerase chain reaction with a lower limit of quantification of 50 copies/mL. Complete EVR (cEVR) was defined as HCV RNA <600 IU/mL at treatment week 12 using the quantitative assay in Step 1. SVR was defined as complete HCV clearance at 24 weeks after treatment cessation.

### IL28B genotyping

Baseline whole blood samples were used for genotyping. We genotyped three SNPs surrounding *IL28B* most strongly associated with response to therapy in the original genome-wide study [15] (rs12979860, rs8099917, and rs12980275) using the iPlex Sequenom platform at the Broad Institute. We obtained a call rate of 97.8% for all three SNPs in our samples. In all ethnic groups, none of the SNPs were out of Hardy-Weinberg equilibrium (*P*>0.001).

### Ethics statement

The A5178 clinical protocol was reviewed and approved by National Institute of Allergy and Infectious Diseases and Food and Drug Administration. All A5178 subjects provided written informed consent. The A5178 protocol and written informed consent was approved by the institutional review boards (IRB) at all participating clinical sites (See supporting information for a full list of participating clinic sites) [30] Subject enrolled in this ancillary study provided written informed consent for genetic testing at recruitment to A5178. This ancillary study was exempt from IRB approval requirement by the Massachusetts General Hospital IRB and was approved by the Broad Institute of MIT and Harvard IRB.

### Statistical Analysis

We performed multivariate logistic regression analysis to test the association between *IL28B* SNPs and cEVR and SVR among subjects with HCV genotypes 1 or 4. Fisher's exact test was used to assess for a statistically significant difference in the proportion of subjects with HCV genotypes 2 or 3 achieving cEVR and SVR in the different *IL28B* genotype groups.

We used logistic regression to develop a prediction score for cEVR and SVR among those with HCV genotypes 1 and 4. We converted continuous variables to ordinal and recoded them so that the higher category predicted cEVR and SVR. We then used forward selection to include variables significantly associated (p<0.05) with these outcomes in a multivariate logistic regression model. Two variables were included (IL28B genotype [three categories] and base-line HCV viral load [three categories]). We then converted the odds ratio estimates of these variables into a point system based on their strength of association with cEVR and SVR and calculated the area under the receiver operating characteristic (ROC) curve for this prediction score. A cut-off score ≤3 was chosen, as it maximized the negative predictive value (NPV) for achieving cEVR and SVR.

The Hosmer-Lemeshow test was used to assess the goodness-of-fit of the predictive models. Statistical significance was set at a two-sided p-value ≤0.05. All statistical analyses were done using SAS version 9.2 software (Cary, NC, USA).

## Results

### IL28B and response to HCV treatment in the HCV/HIV coinfected cohort

**Figure 1:**
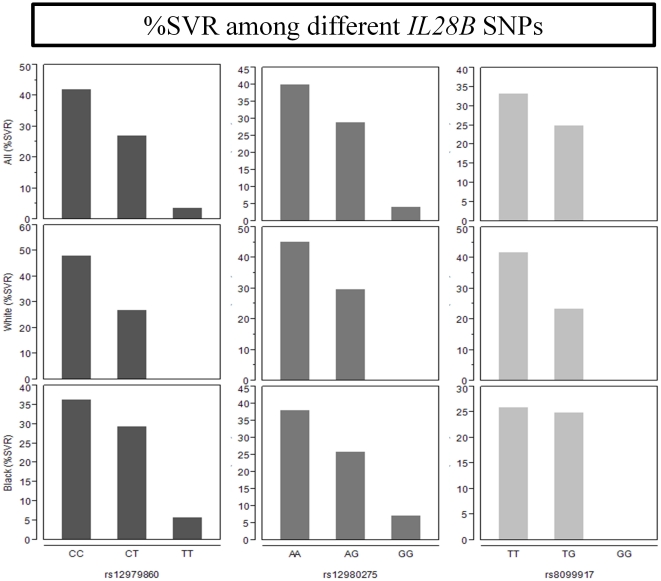
Bar graph showing percent of subjects with different *IL28B* SNPs achieving SVR stratified by ethnicity.

Among 231 HCV/HIV coinfected subjects included in this study (baseline characteristics are shown in Table 1), 197 had HCV genotypes 1 or 4 and 34 had genotypes 2 or 3. Complete EVR (cEVR) and SVR rates among those with genotypes 1 or 4 were 36.5% and 30% respectively. In contrast, cEVR and SVR rates among those with genotypes 2 or 3 were 94%, and 70% respectively. We observed a strong association between *IL28B* genotype and response to therapy among those with genotypes 1 or 4 (Table 2). The odds ratios for cEVR and SVR were 2.98 [1.7–5.3] and 3.4 [1.7–6.9], respectively, with each additional copy of the C allele of rs12979860 (Figure 1). The association between *IL28B* genotype and response to PEG and WBR treatment in this coinfected cohort was independent of age, sex, ethnicity, baseline HCV viral load, or cirrhosis as shown in the multivariate logistic regression model presented in Table 2. In this model both the baseline HCV viral load and *IL28B* genotype emerged as strong and independent predictors of response to PEG/RBV treatment. Furthermore, when adjusting for all three SNPs in a multivariate logistic regression model (model not shown), only rs12979860 was associated with response to HCV treatment, consistent with these SNPs all being in strong linkage disequilibrium with each other.

**Table 1:** Baseline Characteristics of 231 subjects included in our study.

**Age (years)**	47 (SD, 6.9)
**Gender (%)**	
M	83%
F	17%
**Ethnicity (%)**	
White	45%
Black	35%
Hispanic	16%
Other	4%
**HCV Genotype (%)**	
**1**	81%
**2**	10%
**3**	5%
**4**	4%
**HCV baseline viral load (IU/mL)**	4,040,000 [IQR 1,580,000–8,880,000]
**HIV baseline viral load (% undetectable)**	75%
**CD4 count at baseline**	591 (SD, 308)
**Cirrhotic (%)**	14%
**Previous interferon experience (%)**	35%

**Table 2:**
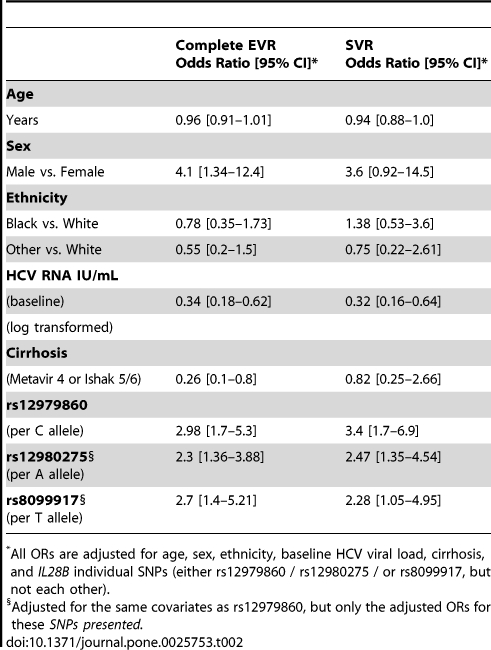
Multivariate logistic regression model for cEVR and SVR among HCV/HIV coinfected subjects with HCV genotypes 1 or 4.

	Complete EVROdds Ratio [95% CI]*	SVROdds Ratio [95% CI]*
**Age**		
Years	0.96 [0.91–1.01]	0.94 [0.88–1.0]
**Sex**		
Male vs. Female	4.1 [1.34–12.4]	3.6 [0.92–14.5]
**Ethnicity**		
Black vs. White	0.78 [0.35–1.73]	1.38 [0.53–3.6]
Other vs. White	0.55 [0.2–1.5]	0.75 [0.22–2.61]
**HCV RNA IU/mL**		
(baseline)	0.34 [0.18–0.62]	0.32 [0.16–0.64]
(log transformed)		
**Cirrhosis**		
(Metavir 4 or Ishak 5/6)	0.26 [0.1–0.8]	0.82 [0.25–2.66]
**rs12979860**		
(per C allele)	2.98 [1.7–5.3]	3.4 [1.7–6.9]
**rs12980275** §(per A allele)	2.3 [1.36–3.88]	2.47 [1.35–4.54]
**rs8099917** §(per T allele)	2.7 [1.4–5.21]	2.28 [1.05–4.95]

* All ORs are adjusted for age, sex, ethnicity, baseline HCV viral load, cirrhosis, and *IL28B* individual SNPs (either rs12979860 / rs12980275 / or rs8099917, but not each other).

§ Adjusted for the same covariates as rs12979860, but only the adjusted ORs for these *SNPs presented.*

A statistically significant association between *IL28B* genotype and response to HCV treatment was not observed among subjects with genotypes 2 or 3 (Table 3a), nor was the responder rs12979860 CC genotype overrepresented in those subjects (Table 3b). A statistically non-significant trend for higher SVR was observed among those with the rs12979860 CC genotype vs. CT/TT (80% vs. 65%) (P = 0.67) (Table 3a). However, the number of subjects with genotypes 2/3 included in our study was likely too small (n = 34) to permit definitive conclusions.

**Table 3:** *IL28B* among HCV/HIV coinfected subjects with HCV genotypes 2 or 3.

**A) Complete EVR and SVR rates among subjects with genotypes 2 or 3**		
**rs12979860**	**cEVR**	**SVR**
**CC**	12/12(100%)	8/10(80%)
**CT + TT**	20/22 (91%)	13/20(65%)
**B) Distribution of ** ***IL28B*** ** rs12979860 genotype in subjects with HCV genotypes 2 or 3 compared to 1or 4**		
**rs12979860**	**Genotype 2 and 3** **(n = 34)**	**Genotype 1 and 4** **(n = 186)**
**CC**	12 (35%)	60 (32%)
**CT + TT**	22 (65%)	126 (68%)

### Ethnic differences in *IL28B* distribution and response to HCV treatment in the HCV/HIV coinfected cohort

Table 4 shows the ethnic distribution of the three *IL28B* SNPs among HCV-HIV coinfected subjects. The responder genotype CC at the rs12979860 locus had a lower frequency among black subjects (17%) as compared to whites (45%) and Hispanics (26%). These ethnic differences were less pronounced for the rs8099917 and rs12980275 SNPs.

**Table 4:** Genotype distribution of the three *IL28B* SNPs in each ethnic group.

	White (103)	Black (80)	Hispanic (38)
**rs12979860**			
**TT**	11%	28%	21%
**CT**	44%	55%	53%
**CC**	45%	17%	26%
**rs8099917**			
**GG**	6%	1%	13%
**TG**	30%	21%	37%
**TT**	64%	78%	50%
**rs12980275**			
**GG**	8%	20%	18%
**AG**	46%	49%	50%
**AA**	46%	31%	32%

Response to PEG and WBR treatment was also lower among black HCV/HIV coinfected subjects as compared to whites with odds ratio of achieving SVR of 0.5 [0.26–0.97] (Table 5). Significant proportions of the lower response rates among blacks were attributed to differences in the distribution of the *IL28B* responder allele at the rs12979860 locus among blacks. Indeed, after adjusting for *IL28B* genotype (rs12979860), the odds ratio for SVR among blacks compared to whites were 0.68 [0.33–1.4] (Table 5).

**Table 5:** Association between black race and SVR unadjusted and adjusted for rs12979860.

	Black vs. White Unadjusted	Black vs. White Adjusted for rs12979860	Percent Change in OR
**SVR**	0.5 [0.26–0.97]	0.68 [0.33–1.4]	36%

### Pretreatment prediction score of the response to PEG/WBR treatment

Given the independently strong association between *IL28B* genotype, baseline HCV viral load, and response to PEG/WBR, we sought to incorporate these variables into a pretreatment prediction score that is capable of identifying coinfected persons with a low a priori likelihood of success with PEG/WBR, so that they may minimize unnecessary exposure to potentially harmful therapy.

**Figure 2:**
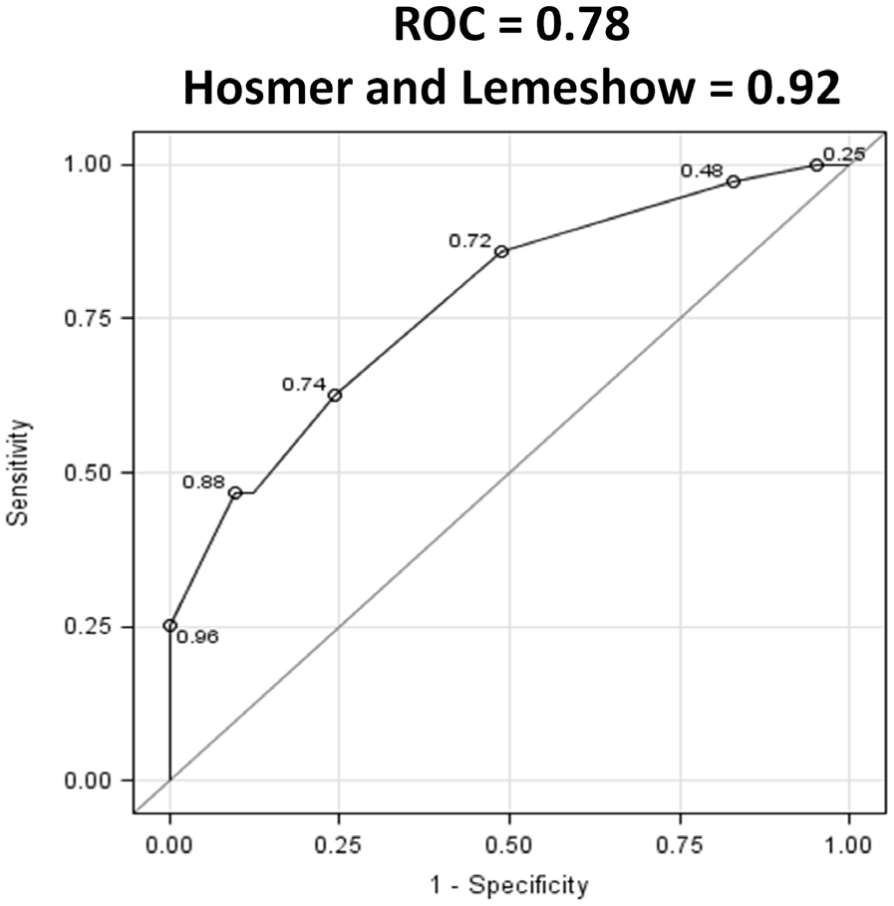
Area under ROC for predicting SVR from a model including *IL28B* genotype (rs12979860) and baseline HCV viral load.

To construct this prediction score, we calculated the area under the ROC for prediction of cEVR and SVR from a model that included *IL28B* genotype (rs12979860) and pretreatment HCV viral load. The area under the ROC for cEVR and SVR was 0.74 and 0.78 respectively (Hosmer–Lemeshow goodness-of-fit test, 0.6 and 0.92) (Figure 2). We then converted odds ratio estimates of these predictors to a simple 12-point system based on their strength of association with cEVR and SVR. The pretreatment NPV for achieving cEVR and SVR for a cut-off score of ≤3 was 88% and 93%, respectively (Table 6).

**Table 6:** Prediction score for complete EVR and SVR.

Predictor	Points	Cut-off Point	Negative Predictive Value (NPV)
**rs12979860**		≤**3 points**	**88% for cEVR** **93% for SVR**
• TT	0		
• CT	3		
• CC	6		
**HCV RNA** IU/mL (base-line)			
• >5,000,000	0		
• 500,000–5,000,000	3		
• <500,000	6		
**Maximum points**	12	**>3** **points**	**Indeterminate**

**Figure 3:**
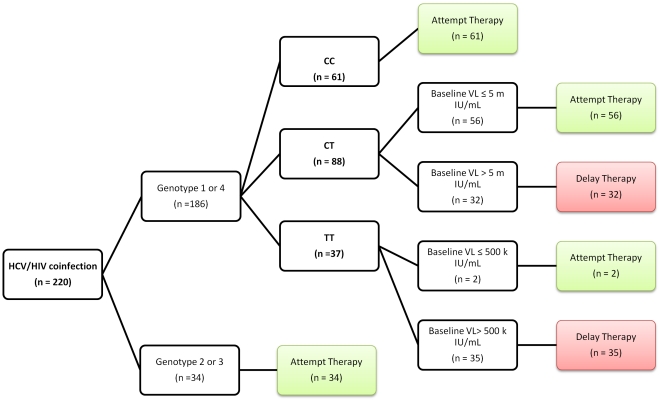
A proposed HCV pretreatment decision tree utilizing our prediction score and its application to the HCV/HIV coinfected cohort.

This prediction score identified 67 coinfected SLAM-C subjects with low likelihood to respond to PEG/WBR HCV treatment (NPV for SVR 93%). Figure 3 shows a proposed pre HCV treatment decision tree utilizing our prediction score and how it applied to our HCV/HIV coinfected cohort.

## Discussion

Since the striking association between SNPs on chromosome 19 surrounding the *IL28B* gene and response to PEG/WBR were reported, multiple confirmatory studies have replicated these findings in cohorts with different racial backgrounds and extended them to subjects with HCV/HIV coinfection. [15], [16], [17], [24], [25], [26], [28] Common limitations to some of these studies were their retrospective nature, which made them susceptible to selection bias and less stringent outcomes ascertainment, and lack of ethnic diversity. [24], [25], [26], [28] In this study we confirmed the association between SNPs surrounding the *IL28B* gene and response to HCV treatment in a prospective multicenter study of HCV/HIV coinfected subjects with different ethnic backgrounds and in whom EVR, cEVR, and SVR were prospectively ascertained in the context of the ACTG A5178 clinical trial.[30] Furthermore, we showed that ethnic differences in the distribution of the *IL28B* SNPs exist between black and white subjects. These differences explain some of the poor PEG/WBR response rates in black coinfected subjects compared to whites.

Contrary to reports from other HCV/HIV coinfected cohorts [24], [25], [26], our study showed that the association between *IL28B* alleles and HCV treatment outcomes is largely additive, where subjects heterozygous for the responder *IL28B* alleles had intermediate HCV treatment success rates compared to those who are homozygote or did not possess a copy of these alleles. A similar trend was recently reported by another study of an HCV monoinfected cohort with advanced liver fibrosis. [21] In addition, a recent study by Dill and colleagues showed that hepatic expression of *IL28B* is decreased in the non-responder (rs12979860) TT genotype as compared to CT or CC [31], a finding previously suggested by another group in peripheral blood. [17] Furthermore, Dill and colleagues showed that differences in interferon stimulated gene (ISGs) expression between those with CT and CC genotypes disappeared after stratification by PEG/WBR treatment response, leading them to conclude that differences in ISG between *IL28B* alleles shown by other groups in unstratified analyses were likely due to unequal assignment of non-responders to the heterozygote *IL28B* group. [32], [33], [34] Although these findings need to be confirmed, they argue that quantification of hepatic ISG expression is a better predictor of SVR among subjects heterozygous for the *IL28B* allele. However, this is not a practical approach in routine clinical practice.

The *IL28B* gene encodes IFN-*λ*3, which along with IFN-*λ*1 and IFN-*λ*2 constitute the type III interferons. HCV RNA in hepatocytes is sensed by pattern recognition receptors, which in turn induce expression of IFN-*λ,* from both plasmacytoid dendritic cells and hepatocytes. When these interferons bind their cognate receptors on hepatocytes and antigen presenting cells in the liver, they upregulate hundreds of ISGs with potent endogenous anti-HCV activity through activation of the Jak-STAT signaling pathway and likely convergence with the type I IFN (IFNα and β) pathway. [19], [35]

An interplay between variable activation of these host endogenous interferon pathways and multiple viral resistance mechanisms, such as amino acid substitutions at positions 70 and 91 of the HCV core protein, accumulation of substitutions in the interferon sensitivity regions (ISDR) of the NS5A protein, and viral recruitment of liver-specific micro RNA (miR-122) to enhance its abundance, may functionally determine the pretreatment HCV viral population and its potential response to exogenous interferon administration.[36], [37], [38], [39], [40] These host and viral factors have been shown to be independent in predicting HCV treatment outcomes [22], [23], [33], and as such, can explain why both the *IL28B* genotype and baseline HCV viral load emerged in our study as strong and independent predictors of PEG/WBR response.

A simple genetic test for the rs12979860 genotype is now commercially available in the United State. [41] However, as shown by us and others, *IL28B* genotype is not perfect in predicting treatment outcomes or completely precluding subjects homozygous for the non-responder alleles from achieving SVR. This is likely due to the complexity of viral-host interactions and variable expression of ISGs even among subjects with favorable *IL28B* genotypes. [31] To that end, we have developed a prediction score capitalizing on the negative predictive value of two strong and consistently reported predictors of HCV treatment outcomes (*IL28B* genotype and baseline HCV viral load).

This simple pretreatment prediction score enabled us to achieve a high negative predictive value for SVR (93%) in this difficult to treat HCV/HIV coinfected cohort. If applied to the SLAM-C cohort prior to HCV therapy, it would have identified 67 subjects with unfavorable pretreatment scores (≤3 points) and minimized their unnecessary exposure to potentially harmful therapy. Furthermore, 58 SLAM-C subjects had less favorable *IL28B* genotypes but low baseline HCV viral load enabling them to achieve a favorable pretreatment prediction score (>3 points), where *IL28B* genotype as a sole measure was not an accurate predictor of their HCV treatment outcome to justify excluding them from therapy based on this measure alone (Figure 3).

Medrano and colleagues have previously derived and validated a prediction score for achieving SVR in predominantly white HCV/HIV coinfected cohort from Spain [28]. The variable included in their prediction score were IL28B genotype rs12979860 (CT or TT vs. CC), liver stiffness assessed by transient elastography (FirboScan), HCV genotype (1 and 4 vs. 2 and 3), and baseline HCV viral load. Their prediction score had a higher area under the ROC than ours (0.892 vs. 0.78). However, this was likely secondary to having a predominantly white cohort, inclusion of HCV genotype as a predictor of outcome, and the inclusion of subjects who only completed a full course of therapy with documented outcomes. Nonetheless, their finding complements our by confirming the importance of *IL28B* genotype and baseline HCV viral load as independent predictors of HCV treatment outcomes.

The strengths of our study include its relatively large sample size, prospective design and outcome ascertainment in the context of a multicenter clinical trial, and inclusion of racial minorities. Several limitations deserve comment. First, SVR was assessed after 72 weeks of therapy. This can be viewed as strength rather than a weakness, since extending the treatment duration would have likely minimized false negative outcomes given the emphasis on the negative predictive value for SVR in our prediction score. Second, about a third of the cohort was interferon experienced. However, this should have had little influence on retreatment outcomes as related to *IL28B* polymorphisms. Furthermore, our derived score predicted null virologic response, an outcome unlikely to change with repeat PEG/WBR treatment. Third, we lacked an appropriate validation cohort to validate our prediction score; thus, appropriate validation is needed before incorporating this score in clinical HCV treatment algorithms. Finally, it is unclear how information about *IL28B* gene polymorphisms will incorporated in HCV/HIV treatment algorithms in the era of direct-acting antiviral (DAA) therapy for HCV; it could be that it would assist triaging patients to conventional vs. DAA therapy to minimize risk for development of DAA resistance.

In conclusion, we have shown that SNPs in regions surrounding the *IL28B* gene are associated with PEG/RBV treatment outcomes in a prospective study of HCV/HIV coinfected persons. These alleles have an additive dose effect and explain some of the racial differences in HCV treatment outcomes between blacks and whites. An HCV pretreatment prediction score that found a high negative predictive value for the *IL28B* non-responder homozygote genotype and high baseline HCV viral load, was able to identify a significant number of coinfected subjects with very low pretreatment likelihood to respond to peg-IFN and RBV. Future approaches to integrate a simple prediction model into the HCV-HIV coinfection management would appear to be warranted to limit potentially harmful exposure to this vulnerable population.
